# A Pilot Study on the Relationship between Primary-School Teachers’ Well-Being and the Acoustics of their Classrooms

**DOI:** 10.3390/ijerph17062083

**Published:** 2020-03-21

**Authors:** Suvi Karjalainen, Jonas K. Brännström, Jonas Christensson, Birgitta Sahlén, Viveka Lyberg-Åhlander

**Affiliations:** 1 Department of Clinical Sciences/Logopedics, Phoniatrics and Audiology, Lund University, 221 85 Lund, Sweden; 2 Saint Gobain Sweden AB, 191 62 Sollentuna, Sweden; 3 Logopedics, Faculty of Arts, Psychology and Theology, Åbo Akademi University, 20 500 Turku, Finland

**Keywords:** well-being, teacher, classroom sound environment, acoustics, vocal health

## Abstract

Although teachers’ well-being and vocal health are affected by noise, research on classroom sound environment from the teachers’ perspective is scarce. This study investigated the relationship between teachers’ well-being and classroom acoustics. The possible influence of teachers’ age, experience, teaching grade and class size on the relationship was also investigated. In this study, well-being refers to self-reported vocal health, stress, burnout and self-efficacy. Twenty-three primary-school teachers answered questionnaires on well-being. In each teacher’s classroom, the acoustical properties were measured with the variables reverberation time, clarity of speech (C_50_) and ventilation system noise (VSN). A series of non-parametric correlations were run to determine the relationship between teachers’ well-being and classroom acoustics. Initially, there was a significant bivariate correlation between burnout and VSN, as well as voice symptoms correlated with VSN and teaching grade. Although the results became not significant after correction for multiple tests, the findings indicate that higher degree of burnout is associated with higher levels of VSN in classrooms, and voice symptoms increase with higher VSN. Teachers working in lower grades had more voice symptoms than those working in higher grades.

## 1. Introduction

Recent reports (2018) have shown that primary-school teachers represent the profession reporting the highest percentage of work-related illness in Sweden [1]. Fifty percent of the teachers experience excessive workload, and 30% feel stressed on an everyday basis [2]. During the last decade, many studies have indicated that voice problems can be viewed as an occupational risk for teachers [3,4,5,6]. Hence, not surprisingly, teaching was the profession with the highest prevalence of self-assessed voice problems in a cohort of 74,351 individuals in a study by Lyberg-Åhlander et al. [4]. Previous studies have indicated a possible relationship between voice problems and decreased general well-being. For example, in the study by Vanhoudt et al. [7], teachers with high scores of self-assessed voice handicap showed a higher relative risk of poorer psychosomatic health. 

In a Swedish study, 15% of the teachers were classified as having high burnout according to the study’s classification system [8]. High burnout scores have been linked to low self-efficacy [9], which is defined as “beliefs in one’s capabilities to organize and execute the courses and actions required to produce given attainments” [10] (p. 3). Higher degree of burnout seems to increase noise annoyance at work amongst pre-school teachers [11]. Teachers often express disturbance by noise and poor room acoustics [12,13]. The degree of disturbance of noise and well-being may well be related to teacher demographics such as teaching experience, teaching grade and the teachers’ age, as well as the number of pupils in class. For instance, longer experience in teaching will likely equip the teacher with more strategies to manage the classroom, which we speculate would have a positive effect both on the atmosphere in the classroom and the teachers’ well-being. In addition to complaints about poor room acoustics, the majority of teachers in the study by Lyberg-Åhlander et al. also felt they were disturbed by the noise caused by pupils and ventilation systems [12]. Teachers with voice problems are more affected by non-beneficial room acoustics than their voice-healthy colleagues are [12,14]. In the study by Ilomäki et al., teachers reported unsatisfactory air quality, stressful work conditions and background noise as most harmful for their voices [15]. In the present study, teachers’ self-reported vocal health, stress, burnout and self-efficacy are measured with questionnaires and presented as “teachers’ well-being”, which is the outcome measure of this study.

A small handful of studies has explored room acoustics from the teacher’s perspective. In the study by Kristiansen et al., an association was seen between teachers’ exposure to noise and their sound pressure level (SPL) of the voice [16]. Exposure to more noise resulted in more vocal symptoms, and increased voice SPL correlated with more cognitive fatigue at the end of the workday [16]. Another of their studies concluded that acoustical refurbishment carried out in classrooms lowered reverberation times and noise levels and made teachers less disturbed by activity noise. However, shorter reverberation times and lower noise levels did not lead to less vocal symptoms or reduced fatigue after work [13]. 

There are different standards for the measurement of classroom acoustics. Traditionally, reverberation time (RT) is used. RT is defined as the time it takes for the sound pressure level (SPL) to drop by 60 dB (ISO 3382-2) [17] after turning off the sound source.

The early sound reflections, within 50 ms, support the voice and are important for speech intelligibility [18]. Late reflections, after 50 ms, will decrease speech intelligibility, making it harder to perceive and understand what the speaker is saying [18]. Traditionally, it has been the listeners’ perspective that has been taken into account when measuring classroom acoustics. A measure for taking the speakers into account—voice support—was developed and described by Pelegrin-Garcia et al. [19]. Our study takes on the speakers’ perspective, but since voice support is difficult to measure, it has been replaced with the measure clarity of speech (C_50_). C_50_ incorporates both early and late reflections and provides an estimate of all sound reflections in the room. C_50_ shows the balance (ratio) between the early and late sound reflections.

In sum, few studies have focused on the speakers’, teachers’, perspective in room acoustics with some exceptions [12,13,14,15,16,19]. Studies exploring the connection between teachers’ well-being, self-efficacy and room acoustics have, to the best of our knowledge, not been published. Therefore, in this paper, we aimed at investigating the relationship between teachers’ well-being (self-reported vocal health, stress, burnout and self-efficacy) and classroom acoustics (RT, C_50_ and ventilation system noise) with the following research questions:

Is there a relationship between teachers’ well-being and their classroom acoustics?

Is such a relationship affected by teachers’ experience in teaching, teaching grade, teachers’ age or class size? 

## 2. Materials and Methods 

In this study, we collected data on primary-school teachers’ well-being and acoustical data from their classrooms. It is a baseline report from a project investigating an intervention program targeting classroom communication and the effects on teachers. The present study is based on pre-intervention data from teachers partaking in a teacher intervention program [20]. The project was approved by the Regional Ethical Review Board in Lund (2016/567). Informed, written consent was obtained from each participant. 

A municipality in southern Sweden invited the research to be conducted in connection to their extensive refurbishment regarding acoustics and lighting in their classrooms. The acoustical refurbishment was adjusted for the individual classrooms, but for a majority of the classrooms, this resulted in the addition of acoustical panels in the ceiling and on the rear wall. The municipality’s school management assisted in choosing the schools and informed the schools’ principals about the project. Schools were chosen to provide approximately the same number of teachers working in already refurbished and yet to be refurbished classrooms. The teachers in the refurbished schools had been working in the refurbished classrooms for a period between two months and two years. Hence, it can be assumed that their answers are based on their current classroom acoustics. The principals approved the project at their schools and informed teachers in grades 3–6 about the project.

This study thus set out to explore the possible co-play between teachers’ well-being and classroom acoustical parameters. Possible effects of lighting conditions have not been further considered in the present study. 

### 2.1. Teachers

Twenty-five primary-school teachers participated; however, two participants were excluded from data analysis because both had filled out only one of the four questionnaires. The 23 teachers (21F/2M) were working in seven different public mainstream schools and had a mean age of 44 (27–63) years and a mean teaching experience of 14.5 (0–31) years. They taught in grades 3–6 (pupils aged 9–11 years) in classes comprising 17 to 37 pupils with a median of 22 pupils. At the point of data collection, school year 2016–2017, the median number of pupils in grades 3–6 in Sweden was 20 pupils [21]. In the municipality investigated, there are more pupils (44%) with a foreign background (defined as born in a country other than Sweden or with two parents born in a country other than Sweden) than the national average (24%) [21]. 

### 2.2. Hearing Screening

All teachers underwent pure tone hearing screening according to ISO 8253-1 [22] at 20 dB HL at 0.25, 0.5, 1, 2, 4, 6 and 8 kHz using a GSI 66 Screening audiometer (Grason Stadler, Eden Prairie, MN, USA) and SA 201 m Sennheiser earphones (Sennheiser electronic GmbH & Co. KG, Wedemark, Germany). Three teachers did not pass and were recommended to consult audiological expertise. However, their hearing impairments were assessed as minor, and these teachers were included in the study. 

### 2.3. Questionnaires 

All the questionnaires are translated from English into Swedish and the VHI-11 is validated in Swedish. Scorings and calculations were made according to the test manuals. The questionnaires were chosen based on official statistics and previous research describing the risk of voice problems, stress and burnout in teachers [1,2,3,4,5,6,8]. Since there is a well-documented relationship between burnout and self-efficacy in teachers [9], a questionnaire targeting self-efficacy was included. There are not many questionnaires in Swedish targeting self-efficacy, hence the choice of Teachers’ Sense of Efficacy Scale: Classroom Management Subscale (TSES). The TSES is translated into Swedish, and it has already been used together with questionnaires for burnout and stress [23]. The questionnaires have previously been used in Karjalainen et al. [20].

*The Voice Handicap Index (VHI-11)* is composed of 11 items measuring self-perceived voice symptoms rated on a five-point, frequency-based scale (0 = never–4 = always). A higher score indicates a higher degree of subjective voice problems (maximum 44 points). The questionnaire has an 12th item, a 100 mm visual analogue scale (VAS), to assess perception of current voice status, where 0 = no voice disorders and 100 = maximum voice disorders [24], Swedish version [25]. 

*Perceived Stress Questionnaire (PSQ)*. PSQ covers 30 items to assess cognitive perceptions of stress. Items are answered on a 4-point frequency-based scale from 1 (almost never) to 4 (almost always). The total sum for all items is used to calculate a PSQ-index ranging from 0 (no stress) to 1 (maximum stress). The scale is general or time-limited (previous month). In this study, the time-limited scale was used [26], Swedish version [23].

*Copenhagen Burnout Inventory (CBI)* consists of 19 items assessing burnout defined as fatigue and exhaustion in relation to specific domains. CBI is divided into three subscales addressing personal (6 items), work-related (7 items) and client-related (6 items) fatigue. Each item has five response options and depending on the item, the response is either frequency-based from 1 (always) to 5 (never/almost never) or to what extent one agrees from 1 (to a very high degree) to 5 (a very low degree). The answers are scored as 1 = 100, 2 = 75, 3 = 50, 4 = 25, 5 = 0. Mean score ranges between 0 and 100 and is calculated both for all the items together and for the three subscales separately, and a higher score indicates a higher degree of burnout [27], Swedish version [28]. In this study, we report the total score for the CBI and not for the three subscales. 

*Teachers’ Sense of Efficacy Scale: Classroom Management Subscale (TSES)* consists of 8 items targeting teachers’ sense of ability to manage the classroom and create pedagogical prerequisites despite distracting events. Responses are given on a 9-point scale from 1 (nothing) to 9 (a great deal). A mean for all items is calculated, and a higher score indicates a greater sense of ability in managing the classroom [29], Swedish version [23].

### 2.4. Measurements

The room acoustics of the teachers’ classrooms were measured for reverberation time T_20_ (RT), clarity (C_50_) and ventilation system noise (VSN) by an acoustician specialized in educational facilities. RT measurements were performed according to ISO 3382-2 [17] and analyzed in octave bands 125 and 250–4 kHz. C_50_ was measured according to ISO 3382-1 [30]. In each room, 12 measurements were made with two different loudspeaker positions. The microphones were positioned in the rear of the room at a distance from loudspeakers >5 m. VSN measurements were carried out in accordance with ISO 10052 [31], which states that each measurement lasts 30 s and the equivalent sound level is given in both dBA (L_A,eq_) and dBC (L_C,eq_) filters. The dBA filter resembles the sensitivity of the human ear, and the dBC filter allows for low frequencies, which are common in VSN, but not always detectable by the human ear. Even though the human ear does not perceive all low frequencies, they nevertheless affect humans [32]. Three consecutive measurements were made in each room. All acoustical measurements were made in unoccupied classrooms, in accordance with the standards.

### 2.5. Statistical Analyses

All statistical analyses were carried out using SPSS (Statistical Package for the Social Sciences, IBM SPSS Statistics 25, Armonk, NY, USA) for Windows. Mann–Whitney U-test was used to analyze differences in both teachers’ well-being and classrooms acoustics between refurbished and non-refurbished classrooms. A series of bivariate correlations were computed with Spearman’s rho to determine the relationship between the teachers’ self-reported well-being and classrooms acoustical measures. The teachers’ well-being variables were VHI-11 (both sum of statements and VAS), PSQ, CBI and TSES and the acoustical variables C_50_, RT (125 Hz and 250–4 kHz) and VSN (dBA and dBC). Thereafter followed bivariate analyses to investigate a possible relationship between the four *demographic variables* teachers’ age, experience, teaching grade and class size and the five variables measuring *teachers’ well-being*. This was followed by the application of Benjamini and Hochberg’s false discovery rate to calculate a corrected alpha level to adjust for multiple tests [33]. Thereafter, non-parametric partial correlations were performed to determine the relationship between the teachers’ well-being measures and classroom acoustical measures whilst controlling for teachers’ age, experience, teaching grade and the number of pupils in class. Since the partial correlations did not yield any significant correlations, no corrections were made. The strength of the correlation coefficient was interpreted using the guideline by Mukaka: negligible (|r| < 0.3), low (0.3 < |r| < 0.5), moderate (0.5 < |r| < 0.7), high (0.7 < |r| < 0.9) and very high (|r| > 0.9) [34]. Correlations > 0.3 that are below the significance level are reported in the Results section. For all analyses, the alpha level was set to *p* ≤ 0.05. Non-parametric statistics were used as the majority of the questionnaires were on an ordinal scale and the sample size was small. 

## 3. Results

Teachers’ responses to the different questionnaires on well-being are presented in Table 1, along with reference values from previous validation studies. 

In Appendix A, descriptive data are presented for the acoustical measures of the 23 (10 refurbished, 13 non-refurbished) classrooms that the teachers worked in. The 23 classrooms had a mean RT 125 Hz of 0.48 s (0.35–0.67); a mean RT 250–4 kHz of 0.43 s (0.30–0.70); and a mean C_50_ of 6.6 dB (2.7–9.2). The mean VSN was 34 dBA (28–42) and 56 dBC (47–61). Recommended limits for RT in school buildings according to SS 25,268 are 0.60 s for 125 Hz and 0.50 s for 250–4 kHz. Since early-reflected sounds increase speech intelligibility, a higher C_50_ value, approximately >6 dB, is beneficial for the pupils’ perceived clarity of speech. For VSN, the recommended limits are 30 dBA and 50 dBC according to SS 25268. 

Ten teachers worked in refurbished classrooms and 13 in non-refurbished. Mann–Whitney U-test showed that well-being did not differ significantly between teachers working in refurbished contra non-refurbished classrooms. Reverberation time, RT 250–4 kHz, was significantly shorter (M = 0.353, Median = 0.365) in refurbished classrooms than in non-refurbished classrooms (M = 0.484, Median = 0.495), *U* = 8.00, *z* = −3.54, *p* = 0.000. C_50_ was significantly higher in refurbished classrooms (M = 8.23, Mdn = 8.15) than in non-refurbished classrooms (M = 5.32, Median = 4.65), *U* = 12.0, *z* = −3.29, *p* = 0.000.

Since the only measures that significantly differed due to refurbishment were RT 250–4 kHz and C_50_, correlations were based on the pooled data from the 23 classrooms to retain more statistical power. 

As for the question of the relationship between teachers’ well-being and the acoustical measures in their classrooms, see streamlined correlation matrix in Table 2, where all measures are shown. 

The bivariate analyses to investigate whether teachers’ well-being was related to teacher demographics (teachers’ age, experience, teaching grade) or class size resulted in one significant correlation, namely, between VHIsum and teaching grade *r_s_*(22) = −0.475, *p* = 0.026. After the same outlier as above was removed, the correlation was *r_s_*(21) = −0.435, *p* = 0.049. However, the non-parametric partial correlations between teachers’ well-being and the room acoustical measures did not result in any significant correlations when controlled for teachers’ age, experience, teaching grade and class size.

Corrections for the bivariate analyses were made to adjust for the multiple correlations calculated in order to get a corrected alpha with Benjamini and Hochberg’s false discovery rate. After corrections, no previously significant correlations remained significant.

## 4. Discussion

This study set out to explore the relationship between teachers’ well-being and classroom acoustics. With respect to the first research question—is there a relationship between teachers’ well-being and their classroom acoustics?—a low significant correlation showed that higher degree of burnout was related to higher ventilation system noise levels (dBA) in classrooms. There was also a relationship between voice symptoms and ventilation system noise (dBA), and after the removal of an outlier, the strength of this relationship increased. It increased from low, non-significant to moderate and significant, indicating that more self-reported voice symptoms were associated with higher ventilation noise levels. As for the second research question—is such a relationship affected by teachers’ experience in teaching, teaching grade, age or the class size?—the negative correlation between voice symptoms (VHIsum) and teaching grade shows that teachers working in lower grades reported more voice symptoms than those working in higher grades. However, when controlling for teachers’ age, experience, teaching grade and class size, we found no significant correlations between teachers’ well-being and room acoustics. Traditionally, research investigating classroom acoustics has been carried out from the listeners’ perspective. The classroom is, however, a workplace where both pupils and teachers need to listen and to talk. The main talker is the teacher. This is one of the few studies investigating the talkers’ situation and well-being in relation to the room acoustics. 

The review on noise exposure and public health by Passchier-Vermeer and Passchier concluded that noise exposure does not only induce hearing impairments but also hypertension and ischemic heart disease, sleep disturbance, annoyance and decreased school performance [37]. Maybe the effects of noise exposure pose an explanation to the correlation in our study between teachers’ higher degree of burnout and higher ventilation system noise. 

Our study, contrary to the study by Rantala and Sala [14], did not find a significant relationship between vocal health and RT. An explanation for this could be that the teachers’ vocal health was good and classroom RTs were close to optimal in our study. Another possible explanation may be that the small sample size was not sufficient to establish a significant relationship. Rantala and Sala [14] used a longer version of the VHI [38] to assess vocal symptoms, and the classroom RTs in their study had a mean of 0.55 s, which is about 0.1 s longer than in our study. They did not find any relationship between VHI scores and room acoustic parameters when their two subgroups were investigated. The subgroups were formed depending on whether the teachers worked in quiet or noisy classrooms. However, when the whole sample was studied, Rantala and Sala found that longer RTs had a positive effect on vocal health [14]. The study by Cantor Cutiva and Burdorf argued for objective measures of classroom acoustics when investigating vocal health in teachers [39]. They included both objective measures and teachers’ self-reports on classroom acoustics and found poor agreement between them. The measured average noise levels were high and RTs long, which may have hampered the possibility to compare teachers in good versus poor room acoustics. There was, however, an association between self-reported poor acoustics and voice symptoms [39]. Pelegrin-Garcia and Brunskog found that teachers with voice problems preferred classrooms with longer RT [40]. However, the length of the RTs has to be carefully considered to balance the interest of the speaker and the listeners. Longer RTs can mask speech and are not beneficial for the pupils’ perception of what is being said [41]. There seems to be a fine line between what helps and what hampers, in the case both of speaking and listening. However, since the two concepts are entwined in most (all) communicatory situations, both need to be taken into account when investigating environments where communication is to take place. Brunskog et al. consequently described a comprehensive concept of “Speakers’ Comfort” [42]. It is defined as “the subjective impression that talkers have when they feel that their vocal message reaches the listener effectively with no or low vocal effort”. Hence, it describes the teachers’ auditive and sensory perception of their own voice, also as it is reflected from the boundaries of the room, and how the teacher perceives the pupils’ perception of the teacher’s message.

Commonly, classroom acoustics are measured with RT and measurements of background noise (e.g., ventilation noise). Moreover, in many standards, RT is evaluated with T_20_. However, comparing classrooms by measuring only T_20_ is not enough due to the nature of the measurement, where the decay of the earliest reflections is not included. The early reflections, within 50 ms, support the voice and are important for speech intelligibility and for speakers’ comfort. When T_20_ is measured, evaluation starts only when the sound pressure level has dropped 5 dB; thus, the early reflections are omitted. Therefore, in the present study, the classroom acoustics were measured with both RT (T_20_) and C_50_. As stated earlier, C_50_ includes early reflections and gives information on all the reflections in the room by showing the ratio between early and late sound reflections. Based on C_50_’s inherent characteristics, it might be hypothesized that good clarity would alleviate the vocal load and be associated with lower burnout due to better speakers’ comfort in getting across to the pupils. We therefore expected relationships between clarity and vocal health and between clarity and burnout. Thus, it was rather surprising that we did not find any significant correlations between teachers’ well-being and clarity (C_50_). A possible explanation for the scarcity of significant results or strong correlations could be that a majority of the present classrooms had good C_50_, even if some of them were not yet refurbished. The teachers’ scores on well-being measures were high (i.e., positive). Thus, the combination of high well-being and near-optimal room acoustics, regarding RT and C_50_, is a likely explanation for the lack of significant associations between these variables. Moreover, the lack of significant results may result from the relatively small sample size. Although our study did not provide statistically supported evidence that C_50_ is part of the room acoustic’s interaction with the speaker, it is still plausible that RT and background noise are not sufficient when investigating the influence of room acoustics from the speakers’ perspective, and more research is needed. More research in this area is warranted.

Recent publications by the Swedish Work Environment Authority [1] and the teachers’ labor union [2] present that the profession with the highest percentage of work-related illness in Sweden is primary-school teachers, whereof 30% state feeling stressed on an everyday basis. Surprisingly, this did not show in our results. To some extent, this might be explained by the small sample size. Moreover, a selection bias might have resulted in the low stress-scores. The teachers who were included in the intervention probably considered that they had time to spare and thought that they themselves would gain something through participation. In addition, through the mere fact that the municipality wherein the schools are situated is taking care of the work environment gives the teachers a sense of support. 

Only one (TSES) out of the four questionnaires in our study is validated on teachers specifically. This should be considered, as it might be a part of an explanation for the low rating of voice symptoms, burnout and stress. 

Despite the sparse evidence about the relationship between teachers’ well-being and classroom acoustics, we argue that teachers’ well-being in their working environment affects the way they work and communicate in the classroom. The quality of classroom communication is fundamental for pupils’ learning. However, the physical classroom environment might not only challenge teachers’ well-being but also pupils’ learning. Background and activity noise hamper classroom communication, and the acoustical design of the classroom does not always suit today’s classroom activities, which might have a negative effect on pupils’ performance in the classroom [43,44,45]. This is an important aspect to explore further. It is of utmost importance to support teachers’ vocal and general well-being and pupils’ listening and communicatory conditions to enable good-quality learning. 

## 5. Conclusions

This study is one of few focusing on the speakers’, i.e., teachers’, well-being in relation to the classroom acoustics. This study indicates that higher degree of burnout in teachers is associated with higher levels of VSN in classrooms. Moreover, teachers’ voice symptoms increase with higher VSN. Teachers working in lower grades had more voice symptoms than those working in higher grades. 

## Figures and Tables

**Table 1:** Self-report questionnaires targeting teachers’ well-being presented with number of responding participants (*n*), mean (M) and standard deviation (SD). Reference values from previous validation studies (some based on clinical populations) are also shown.

Measure	*n*	M	SD	Values from Validation Studies
The Voice Handicap Index (VHI11-sum)	23	1.17	1.72	Patients: M = 12.96, SD = 9.70 Controls: M = 2.11, SD = 2.52 [25]
The Voice Handicap Index (VHI11-VAS)	21	1.04	1.30	Patients: M = 4.38, SD = 3.12 Controls: M = 1.43, SD = 1.98 [12]
Perceived Stress Questionnaire (PSQ index)	23	0.28	0.13	Low stress: ≤ 0.34, Moderate stress: >0.34–≤0.46, High stress: > 0.46 [35]
Copenhagen Burnout Inventory (CBI)	22	28.11	12.60	M = 33.3, SD = 17.3 High degree of burnout > 50 [27]
Teachers’ Sense of Efficacy Scale: subscale classroom management (TSES)	23	7.76	0.70	Range of mean from five different countries: M = 6.59–7.65 [36]

**Table 2:** Streamlined correlation matrix for the bivariate correlations computed with Spearman’s rho between the well-being measures and acoustical measures.

Acoustical Measures	VHI11-VAS	VHI11-Sum	PSQ Index	CBI	TSES: Subscale Classroom Management
VSN dBA	0.123	0.507 *^,1^	0.023	0.437 *	−0.225
VSN dBC	−0.035	−0.277	−0.111	0.160	0.076
RT 125Hz	−0.150	−0.095	0.217	0.006	−0.253
RT 250–4 kHz	−0.325	−0.135	0.120	−0.003	−0.197
C_50_	0.275	0.180	−0.017	0.100	0.110

* Correlation is significant at the 0.05 level (2-tailed). ^1^ (VHIsum) had an outlier; the correlation in the matrix is with the outlier removed.

## Appendix A

Acoustical measures and respective values for *n* = 23 non-refurbished (*nr*) and refurbished (*r*) classrooms. Different letters A to G, represent different schools and numbers the different classrooms within the same school.

**Table:**

Classroom	C_50_	RT 125 Hz	RT 250–4 kHz	VSN dBA	VSN dBC
A1*r*	8.6	0.35	0.35	33	56
A2*r*	7.2	0.55	0.40	31	56
A3*r*	7.9	0.48	0.36	33	60
A4*r*	7.4	0.49	0.37	32	61
B1*nr*	7.6	0.46	0.37	40	59
B2*nr*	6.6	0.64	0.39	35	56
C1*nr*	4.6	0.62	0.53	35	55
C2*nr*	4.2	0.67	0.53	33	56
C3*nr*	4.7	0.61	0.50	33	55
C4*nr*	4.0	0.62	0.52	33	57
C5*nr*	4.2	0.52	0.54	36	61
C6*nr*	4.9	0.49	0.48	35	58
D1*nr*	9.2	0.36	0.38		
E1*nr*	5.3	0.37	0.44	36	57
E2*nr*	6.7	0.38	0.42	38	58
E3*nr*	4.5	0.42	0.49	33	53
F1*nr*	2.7	0.59	0.70	29	47
G1*r*	8.9	0.44	0.31	42	54
G2*r*	9.1	0.37	0.30	38	53
G3*r*	8.7	0.41	0.31	39	54
G4*r*	8.0	0.44	0.40	33	56
G5*r*	8.2	0.42	0.35	28	54
G7*r*	8.3	0.42	0.38	31	56
